# Supplementary material for: The Toxicity of Salicylhydroxamic Acid and Its Effect on the Sensitivity of Ustilaginoidea virens to Azoxystrobin and Pyraclostrobin
Source: J Fungi (Basel). 2022 Nov 21;8(11):1231. doi: 10.3390/jof8111231 (PMC9692728; doi:10.3390/jof8111231)
Supplement: Supplementary file 1 [file jof-08-01231-s001.zip › jof-2025394-supplementary.pdf]

Table S1. Effects of SHAM at 10 μg/mL on EC_{50} values of azoxystrobin against mycelial growth of *U. virens* on potato sucrose agar (PSA) and minimal medium (MM).

**Isolate**	**EC_{50} (μg/mL) on PSA**		Fold change	EC_{50} (μg/mL) on MM		Fold change
	**Without SHAM**	**With SHAM**		Without SHAM	With SHAM	
GL18	0.027	0.033	0.82	0.124	0.084	1.48
GY20	0.089	0.053	1.68	0.120	0.009	13.33
HWD	0.251	0.148	1.70	0.198	0.039	5.08
JR4	0.074	0.083	0.89	0.128	0.008	16.97
JS28	0.291	0.189	1.54	0.154	0.015	10.27
JS60	0.129	0.117	1.10	0.056	0.020	2.80
YD2	0.279	0.108	2.58	0.141	0.042	3.33
YZ10	0.126	0.096	1.31	0.119	0.015	8.01
ZJ4	0.233	0.063	3.70	0.294	0.035	8.34
ZJ26	0.051	0.042	1.21	0.019	0.009	2.06
Average	0.155	0.093	1.67	0.135	0.028	4.82
Paired *t* test	$P = 0.020$			$P < 0.001$		

Table S2. Effects of SHAM at 10 μg/mL on EC_{50} values of pyraclostrobin against mycelial growth of *U. virens* on potato sucrose agar (PSA) and minimal medium (MM).

**Isolate**	**EC_{50} (μg/mL) on PSA**		Fold change	EC_{50} (μg/mL) on MM		Fold change
	**Without SHAM**	**With SHAM**		Without SHAM	With SHAM	
GL18	0.092	0.013	7.08	0.108	0.015	7.20
GY20	0.084	0.052	1.62	0.038	0.035	1.09
HWD	0.046	0.031	1.48	0.081	0.019	4.26
JR4	0.099	0.014	7.07	0.048	0.006	8.00
JS28	0.025	0.018	1.39	0.056	0.007	8.00
JS60	0.033	0.018	1.83	0.039	0.012	3.25
YD2	0.118	0.032	3.69	0.112	0.009	12.44
YZ10	0.063	0.022	2.86	0.088	0.007	12.57
ZJ4	0.083	0.007	11.86	0.078	0.009	8.67
ZJ26	0.058	0.042	1.38	0.123	0.045	2.73
Average	0.070	0.025	2.80	0.077	0.016	4.81
Paired *t* test	$P < 0.001$			$P < 0.001$		
